# Anatomical Variations in Aortic Arch Branching Pattern: A Computed Tomography Angiography Study

**DOI:** 10.7759/cureus.36731

**Published:** 2023-03-27

**Authors:** Rabia Tasdemir, Ömer Faruk Cihan, Rümaysa Ince, Fatma Sevmez

**Affiliations:** 1 Department of Anatomy, School of Medicine, Gaziantep Islam Science and Technology University, Gaziantep, TUR; 2 Department of Anatomy, School of Medicine, Gaziantep University, Gaziantep, TUR

**Keywords:** morphology, variation, computed tomography angiography, branching pattern, aortic arch

## Abstract

Introduction: Variations in the branching pattern of the aortic arch (AA) are common. Modification of intravascular stents should be considered taking into account these AA branching variations. Identification of supra-aortic branching types and frequencies is important for specialists planning surgery in this region. In endovascular interventions to the AA, aortic stent grafts should be modified according to the variations of the branching patterns of the AA. In any surgical intervention to the region where the supra-aortic branches are located, ignorance of the variations may cause unwanted injuries or complications.

Methods: In this study, 699 computed tomography angiography (CTA) images were reviewed to investigate AA branching variations using the Horos software (an open-source image viewer). Four groups were constructed based on the number of branches emerging from the aortic arch, which were further divided into subtypes.

Results: A total of 699 CTA images from 320 males and 379 females were included in this study. The usual AA branching pattern (type 3b1) was found in 68.5% of the patients. The combined prevalence of other eight branching patterns, designated as variations, was 31.5%. Variation types 1b1, 3b2, and 4b5 were identified in one patient each. Overall, types 2b1 and 2b2 had a prevalence of 28.3%. The type 2b3 variation was observed in 1.6% of the patients. The least common variations were type 4b1 (0.7%) and type 3b2 (0.1%).

Conclusion: The identification of variations in AA branching patterns by CTA prior to surgical or endovascular interventions involving the aortic arch is important. Thus, specialists planning interventions in this region need to be aware and have knowledge of atypical aortic branching patterns. Higher prevalence rates of AA branching patterns compared to previous studies were identified in the Turkish population in this study and therefore, a comprehensive, multicenter study is needed to determine the cause of this differential finding.

## Introduction

The aorta is the main artery of the body and after originating from the heart, it continues as the ascending aorta, aortic arch and descending aorta (thoracic and abdominal) [1-3]. The arteries branching off from the aortic arch (AA) supply blood to the head and neck and upper extremities [1,2,4]. The usual branching pattern of AA, from right to left, is the brachiocephalic trunk (later divided into the right subclavian artery (RSA) and right common carotid artery (RCCA)), left common carotid artery (LCCA) and left subclavian artery (LSA) [2,3,5-7]. This type of classic branching is seen in 49.7-94.3% of the population [2,7]. However, some variations may occur in the branching pattern of AA during embryologic development [3,4]. Between the fourth and eighth weeks of the fetal period, some of the supra-aortic branches originating from the aortic sac are absorbed into the aortic sac. Depending on the extent of this process, this results in an increase or decrease in the number of branches [1,3,7]. 

These variations are usually asymptomatic and are discovered incidentally during radiological imaging, autopsy, and dissection [2,7]. On the other hand, anatomical variations in the branching pattern of the aortic arch may result in symptoms such as dysphagia and dyspnea as well as various clinical problems [5,7-10].

In cases requiring endovascular aortic arch repair, aortic stent grafts need to be modified taking into account the variations in AA branching pattern [11]. At the same time, detailed understanding of the branching pattern of AA is of utmost importance for the prevention of complications that may occur in radiological or surgical interventions to the supra-aortic branches and for successful performance of the procedure [1,7]. The identification of the anatomic variations of AA branching pattern and knowledge of their frequencies in the population are essential while planning thoracic and neck surgeries and endovascular aortic interventions [12].

In this study, we aimed to determine the frequency of the anatomical variations of the aortic arch branching pattern on computed tomography angiography (CTA) images retrospectively and to analyze the results by sex. In doing so, we aimed to contribute to the literature by providing relevant data for use by interventional radiologists and cardiovascular and neck and thoracic surgeons.

## Materials and methods

Ethical approval dated 13.07.2021 and numbered 2021/37 was obtained from the Ethics Committee for Non-Invasive Clinical Research of Gaziantep Islam Science and Technology University.

This study was carried out retrospectively on the archived images of patients who underwent CTA imaging for various reasons at Gaziantep University Faculty of Medicine between 2017 and 2021. Approximately 2000 CTA images were identified through archive screening, among which a total of 699 images that best suited our purpose were selected.

The exclusion criteria were poor image quality due to motion artifacts or inadequate distribution of the contrast agent, absence of the aortic arch and its branches in the field of view, and prior surgery or endovascular treatment for the aortic arch. Of 699 images, 379 were from females and 320 were from males. The mean age of the patients was 55.78 ± 21.13 years (range: 1-94 years). As variations of the AA branching patterns occur during embryological development, the age restriction was not applicable. The images were reviewed by two independent specialists. The data were anonymized to avoid the identification of the patients.

Patient images were acquired using a 64-detector CTA (VCT XTe Light Speed; Milwaukee, WI: General Electric Company). CTA parameters were as follows: collimation, 40 mm (64 x 0.625); rotation time, 0.35 s; pitch value, 1; x-ray tube voltage, 100-120 kV and 150-600 mA; detector thickness, 0.625-2 mm and reconstruction interval 0.625-2 mm.

From the two-dimensional (2D) CTA images, three-dimensional (3D) reconstructions were obtained using the Horos (https://horosproject.org) software (an open-source image viewer). After transferring the images to the Horos software, the bone and other anatomical structures around the aorta were removed manually on the software.

The CTA images of 699 patients with exposed aorta were reviewed to identify the branching pattern of AA. Based on the systematic classification described by Natsis et al., subtypes were modified according to results and represented within four groups the number of branches emerging from the AA [13]. In addition, the branching types identified were analyzed separately to determine their individual prevalence.

Typing of AA branching patterns based on the number of emerging branches

**Figure 1:**
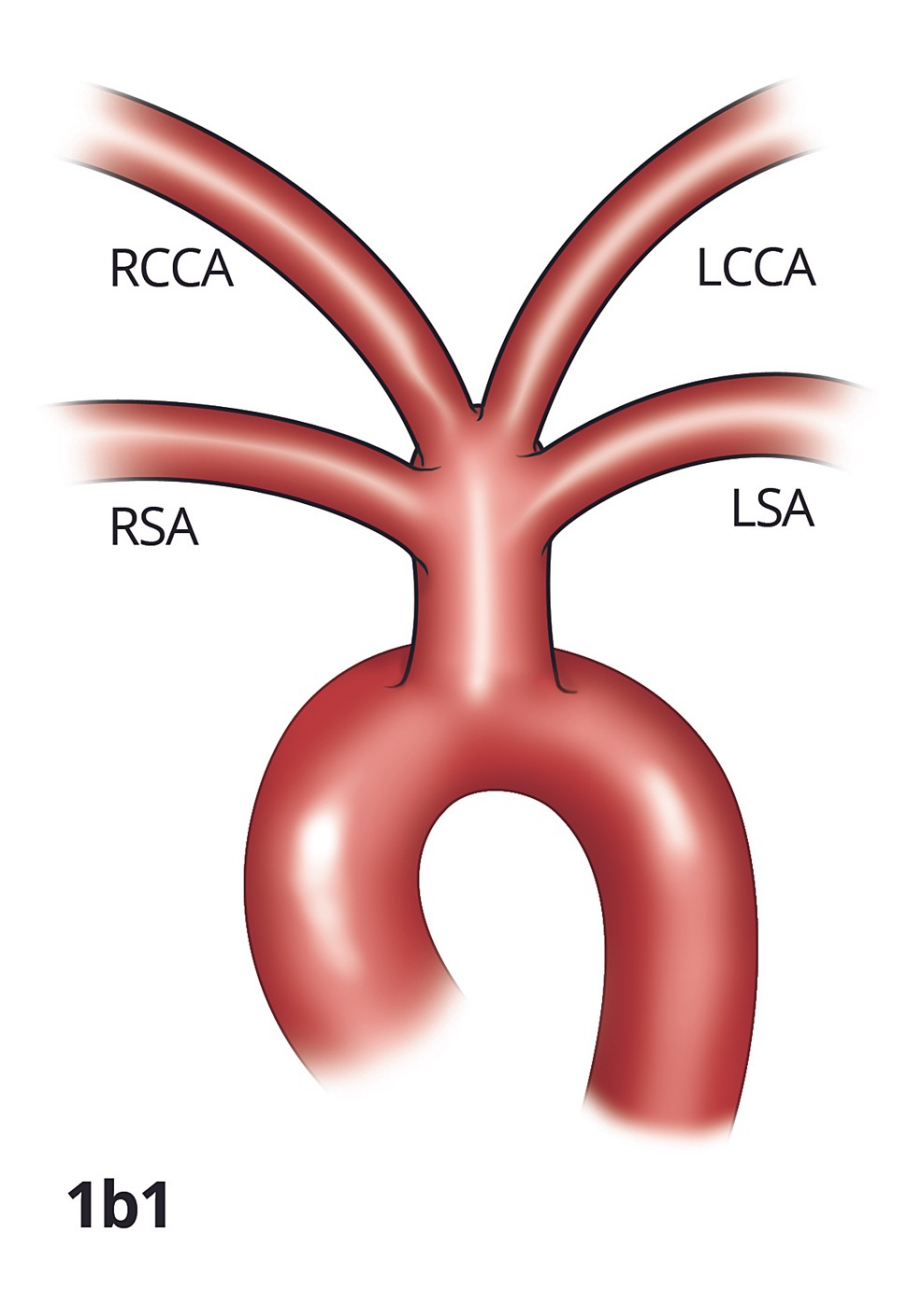
Schematic representation of supra-aortic branches arising from a single root The image is created by the authors of this study. RSA: right subclavian artery; RCCA: right common carotid artery; LCCA: left common carotid artery; LSA: left subclavian artery

Those emerging from the AA as a single trunk were classified as follows (Figure 1): 1b1 - the right subclavian artery (RSA), right common carotid artery (RCCA), left common carotid artery (LCCA), and left subclavian artery (LSA) branch off from a common trunk.

**Figure 2:**
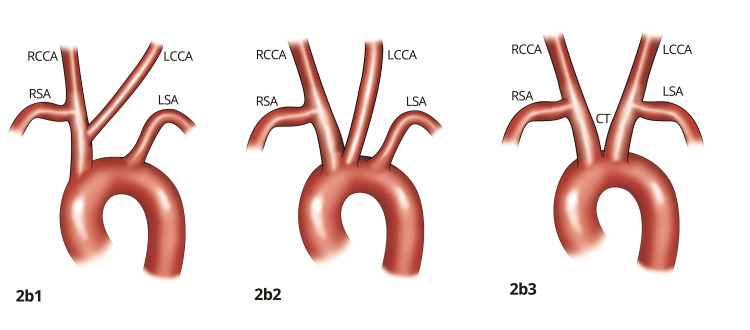
Subgroups of branching that originate from the aortic arch as two branches The image is created by the authors of this study. RSA: right subclavian artery; RCCA: right common carotid artery; LCCA: left common carotid artery; LSA: left subclavian artery; CT: common trunk

Those arising from the AA as two branches were classified as a separate group as follows (Figure 2): 2b1 - a branch and LSA originate from the aortic arch. From the branch, RCCA, RSA, and LCCA emerge. 2b2 - the brachiocephalic trunk (BCT) and LCCA arise from the aortic arch at very close origins and LSA branches off from the aortic arch. 2b3 - two branches come off from the aortic arch. RCCA and RSA arise from one of the branches, and LCCA and LSA from the other branch.

**Figure 3:**
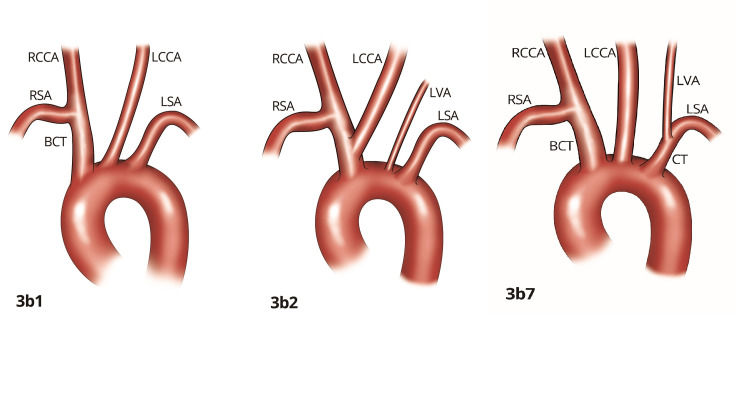
Subgroups of branching that originate from the aortic arch as three branches This image is created by the authors of this study. BCT: brachiocephalic trunk; RSA: right subclavian artery; RCCA: right common carotid artery; LCCA: left common carotid artery; LSA: left subclavian artery; LVA: left vertebral artery; CT: common trunk

Those arising from the AA as three branches were classified as follows (Figure 3): 3b1 - BCT, LCCA, and LSA arise from the aortic arch. RCCA and RSA emerge from the BCT. 3b2 - BCT, left vertebral artery (LVA), and LSA branch off from the aortic arch. RCCA, LCCA, and RSA originate from the BCT. 3b7 - BCT, LCCA, and another branch come off from the aortic arch. RCCA and RSA arise from the BCT. LVA and LSA arise from the other branch.

**Figure 4:**
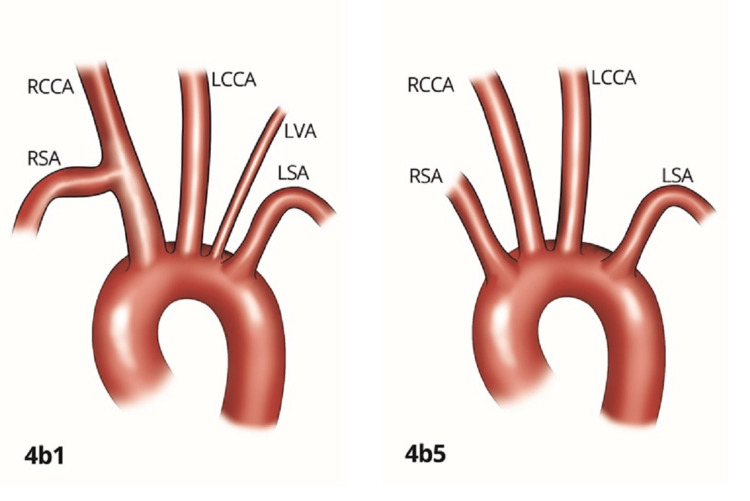
Subgroups of branching that originate from the aortic arch as four branches This image is created by the authors of this study. RCCA: right common carotid artery; RSA: right subclavian artery; LCCA: left common carotid artery; LSA: left subclavian artery; LVA: left vertebral artery

Those arising from the aortic arch as four branches were classified as follows (Figure 4): 4b1 - BCT, LCCA, LVA, and LSA emerge from the aortic arch. 4b5 - RSA, RCCA, LCCA, and LSA come off from the AA as separate branches.

Statistical analysis

Descriptive statistics of the study data were presented as frequency and percentage values. Chi-squared test was used to examine the relationship between sex and variations in the AA branching pattern. Statistical analyses were conducted using SPSS version 22.0 (Armonk, NY: IBM Corp.). A p<0.05 was considered statistically significant.

## Results

A total of 699 images, 320 males and 379 females, were included in this study. The mean age of the patients was 55.78±21.13 years. The frequencies of respective branching variations were identified as discussed below.

1b1

**Figure 5:**
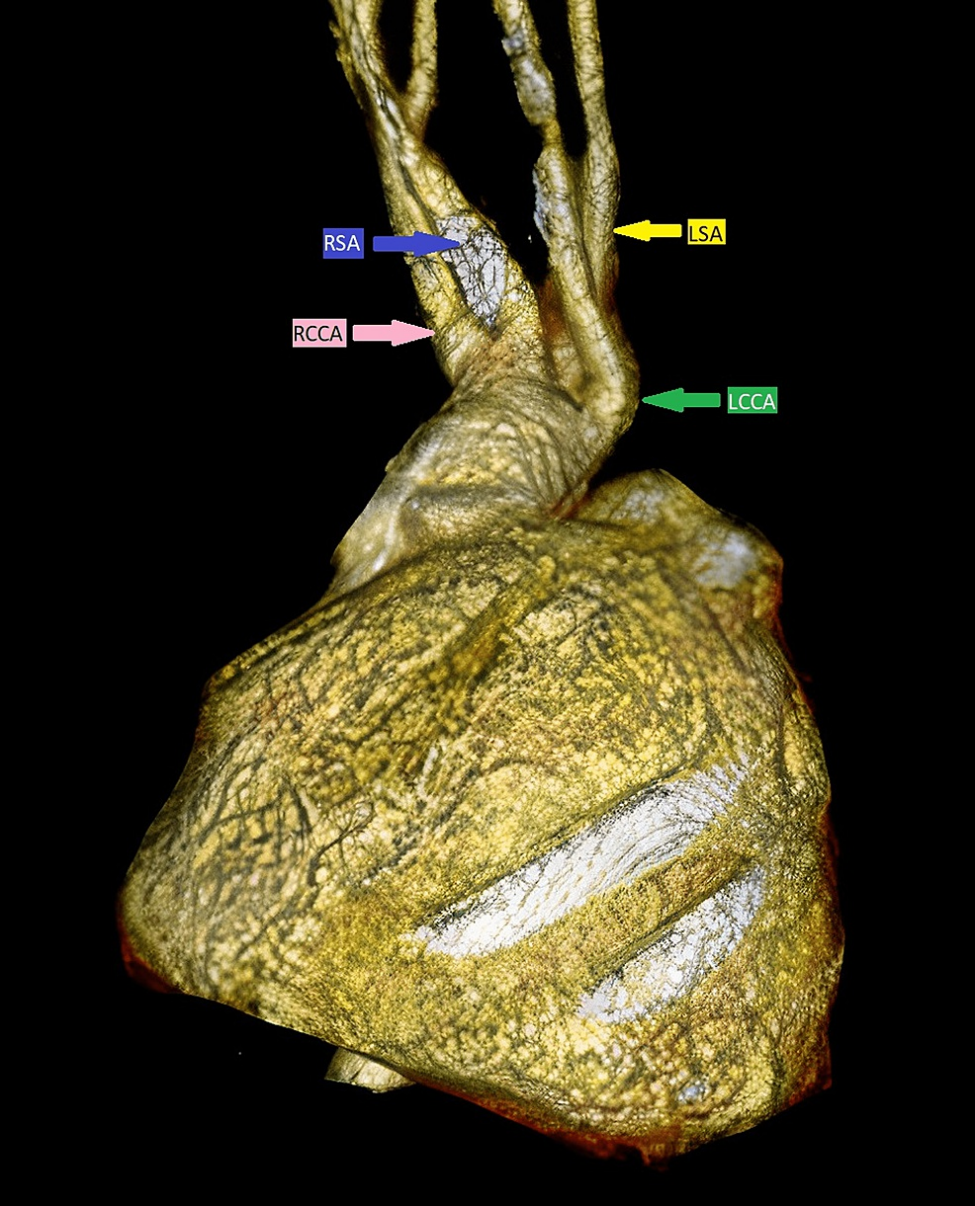
CTA image of aortic arch and supra-aortic branches belonging to group 1b1 CTA: computed tomography angiography; LSA: left subclavian artery; LCCA: left common carotid artery; RCCA: right common carotid artery; RSA: right subclavian artery

RSA, RCCA, LCCA, and LSA branch off from a common trunk (CT). This type of branching was seen in only one (0.1%) patient. This was a female patient (Figure 5).

2b1

**Figure 6:**
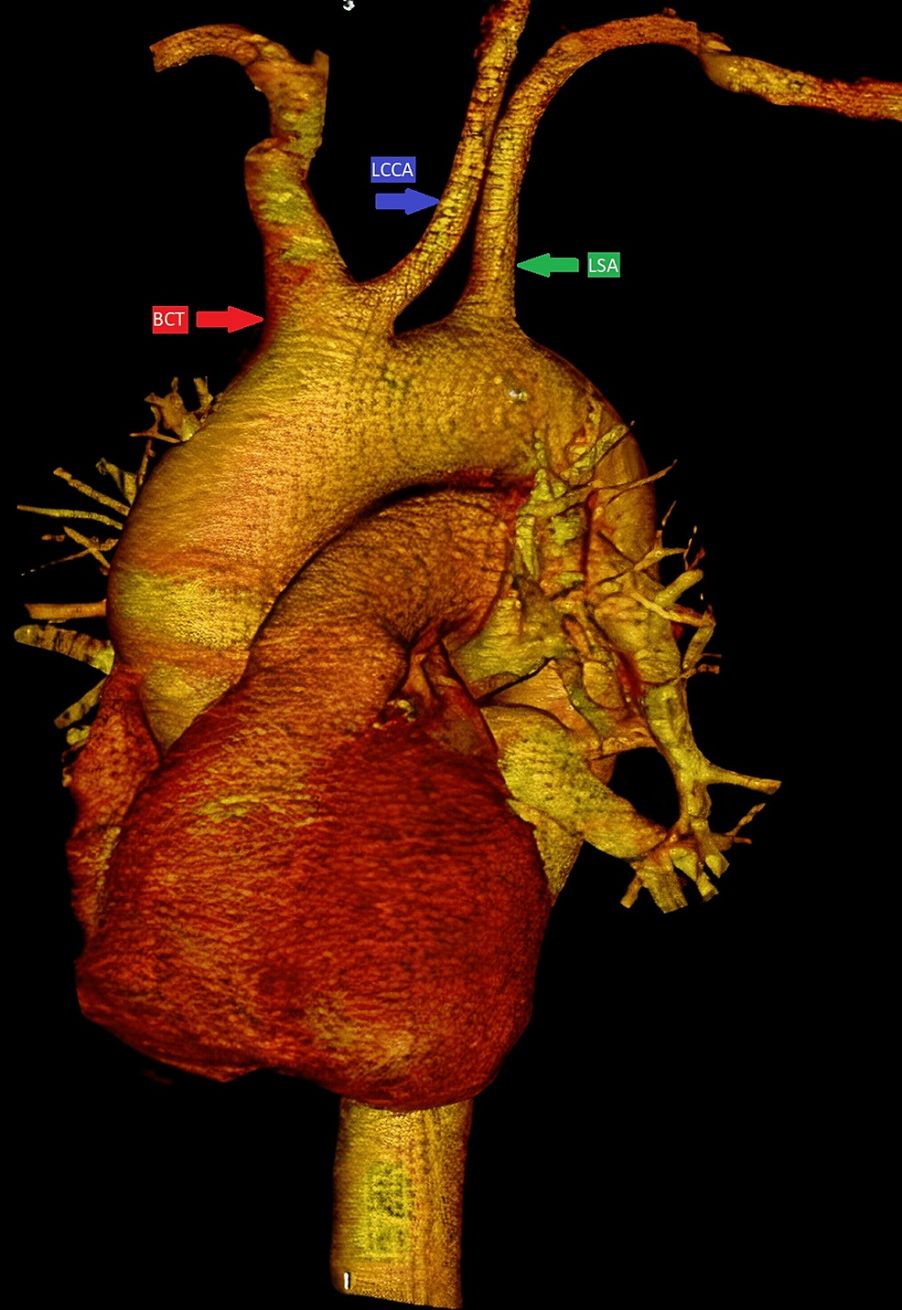
CTA image of aortic arch and supra-aortic branches belonging to 2b1 subgroup CTA: computed tomography angiography; LSA: left subclavian artery; LCCA: left common carotid artery; BCT: brachiocephalic trunk

Two branches come off from the AA, with a common trunk on the right and LSA on the left. RSA, RCCA, and LCCA branch off from the common trunk. This type of variation was detected in 168 (24%) patients, including 60 males and 108 females (Figure 6).

2b2

**Figure 7:**
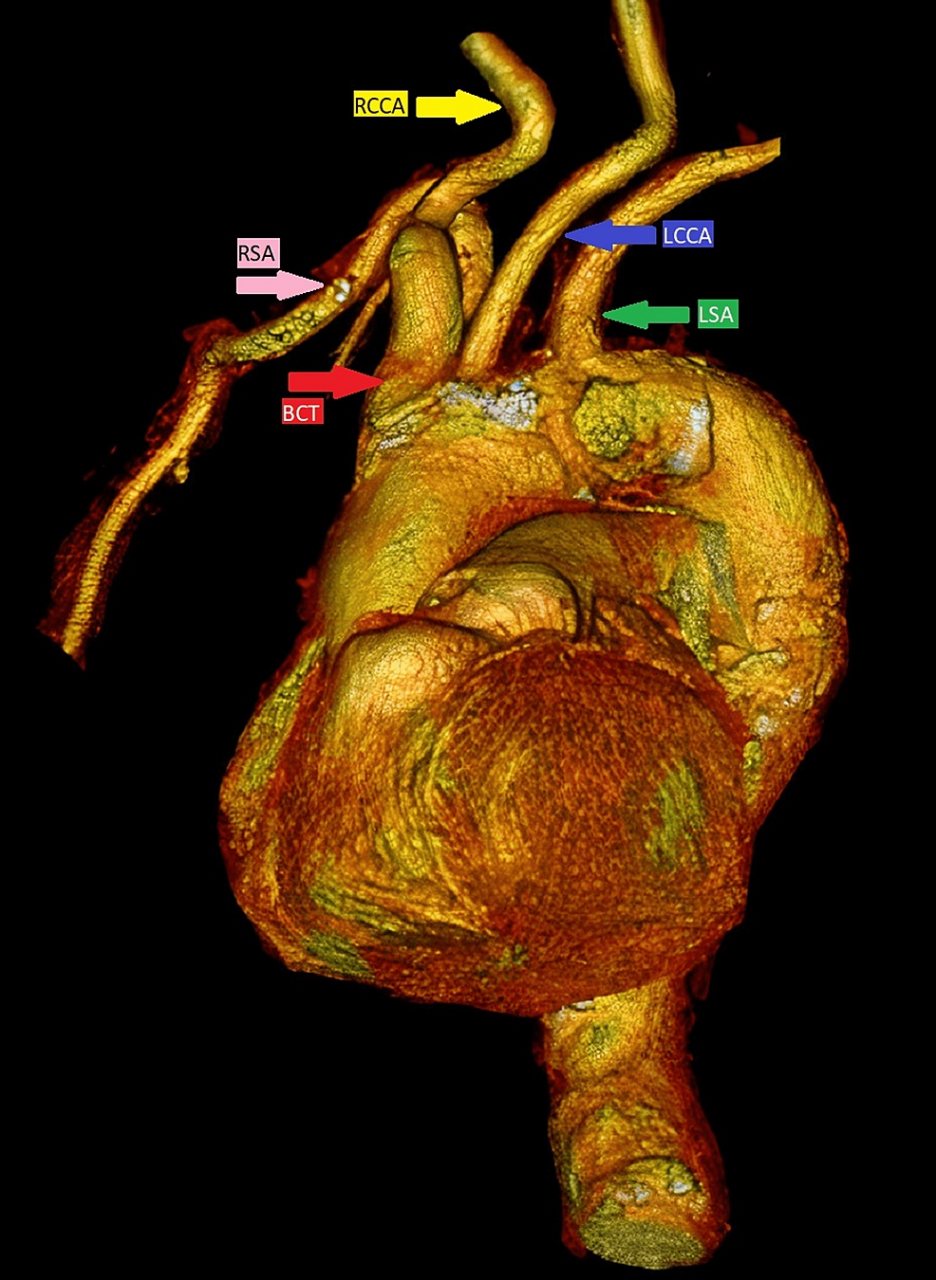
CTA image of aortic arch and supra-aortic branches belonging to 2b2 subgroup CTA: computed tomography angiography; RSA: right subclavian artery; RCCA: right common carotid artery; LCCA: left common carotid artery; LSA: left subclavian artery; BCT: brachiocephalic trunk

AA, BCT, and LCCA come off from a common ostium (V-shaped). LSA arises from a different origin. This type of branching was observed in a total of 30 (4.3%) patients, including nine males and 21 females (Figure 7).

2b3

**Figure 8:**
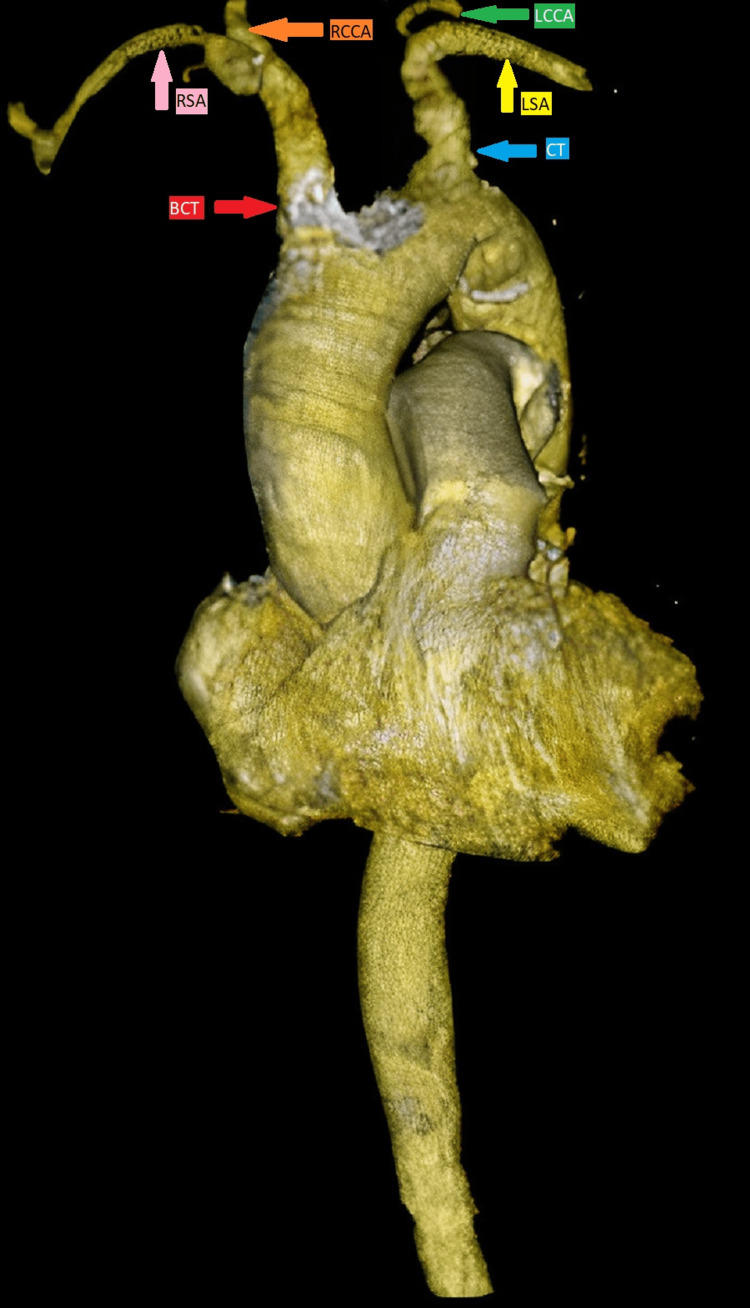
CTA image of aortic arch and supra-aortic branches belonging to 2b3 subgroup CTA: computed tomography angiography; CT: common trunk; RSA: right subclavian artery; RCCA: right common carotid artery; LCCA: left common carotid artery; LSA: left subclavian artery; BCT: brachiocephalic trunk

In this type of variation, BCT and a common trunk (CT) originate from the AA. LCCA and LSA branch off from the common trunk. Eleven (1.6%) patients (two males, nine females) showed this type of variation (Figure 8).

3b1

**Figure 9:**
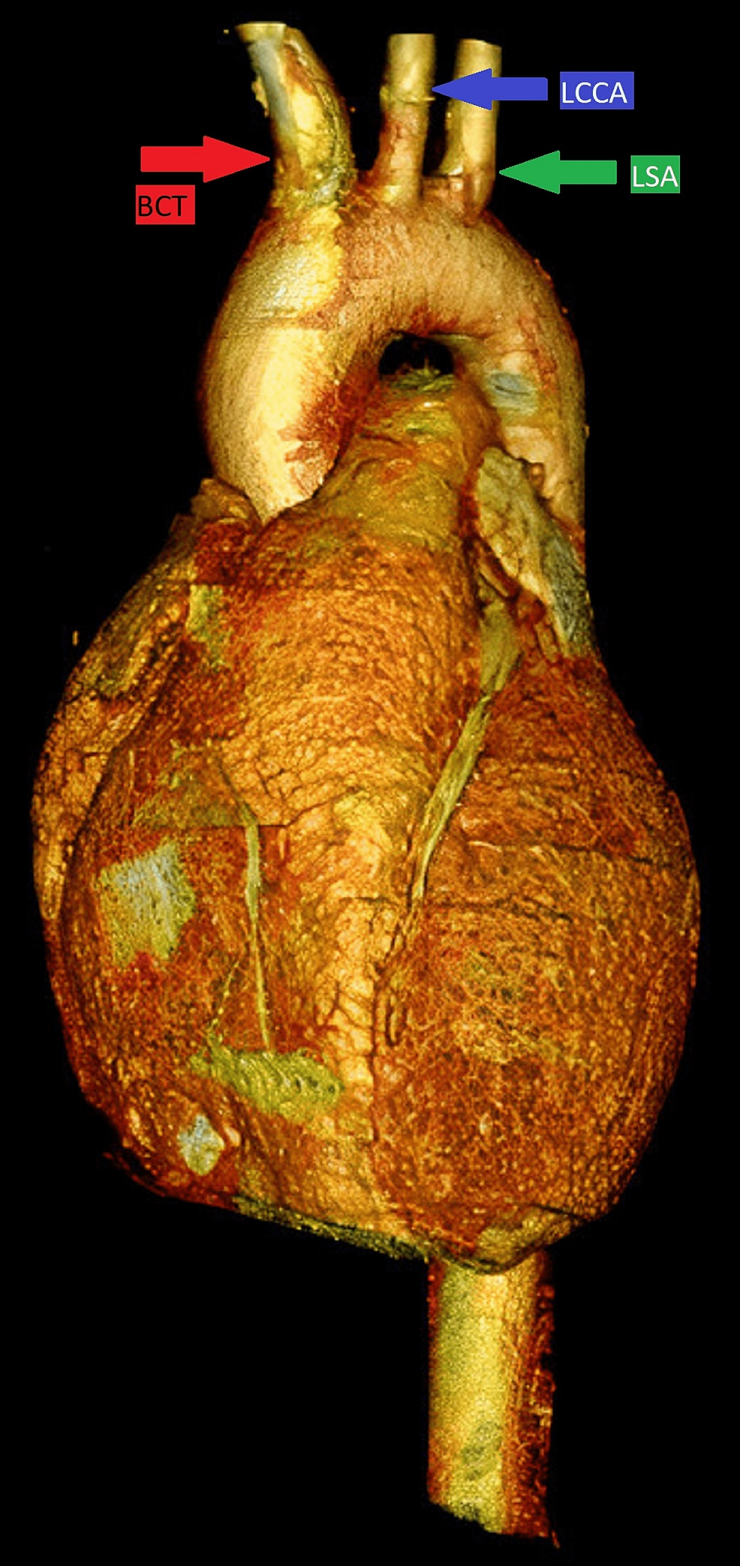
CTA image of arcus aorta and supra-aortic branches belonging to 3b1 subgroup (the normal branching pattern) CTA: computed tomography angiography; LCCA: left common carotid artery; LSA: left subclavian artery; BCT: brachiocephalic trunk

This type represents the normal branching pattern. From right to left, three branches, BCT, LCCA, and LSA, come off from the AA, respectively. This usual branching pattern was identified in a total of 479 (68.5%) patients, including 244 males and 235 females (Figure 9).

3b2

**Figure 10:**
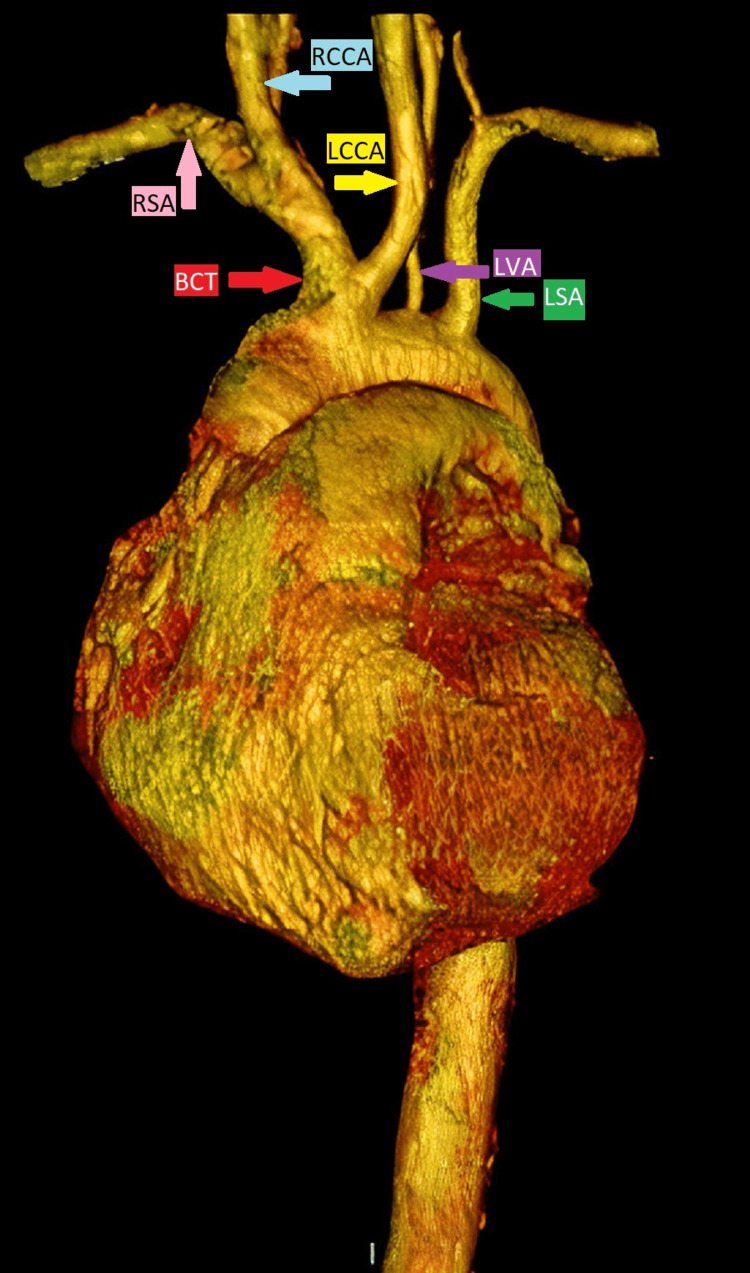
CTA image of arcus aorta and supra-aortic branches belonging to 3b2 subgroup CTA: computed tomography angiography; CT: common trunk; RSA: right subclavian artery; RCCA: right common carotid artery; LCCA: left common carotid artery; LSA: left subclavian artery; LVA: left vertebral artery

AA, BCT, LVA, and LSA emerge as three branches. LCCA, RCCA, and RSA branch off from the AA. This type of variation was seen in only one (0.1%) patient. This was a male patient (Figure 10).

3b7

**Figure 11:**
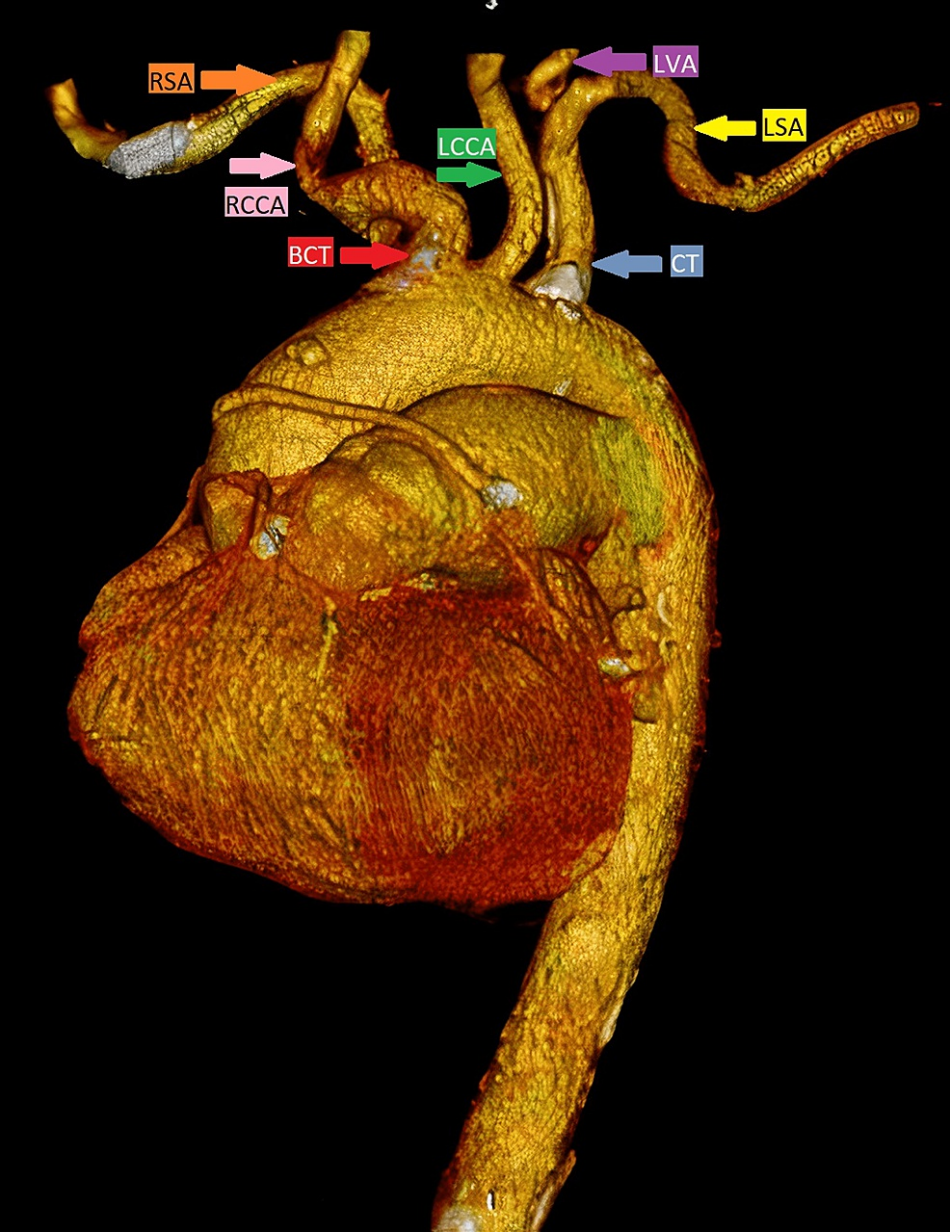
CTA image of arcus aorta and supra-aortic branches belonging to 3b7 subgroup CTA: computed tomography angiography; CT: common trunk; RSA: right subclavian artery; RCCA: right common carotid artery; LCCA: left common carotid artery; LSA: left subclavian artery; BCT: brachiocephalic trunk; LVA: left vertebral artery

AA gives rise to BCT, LCCA, and a common branch. LVA and LSA originate from the common branch. This branching pattern was observed in only three (0.4%) patients. All patients with this variation were female (Figure 11).

4b1

**Figure 12:**
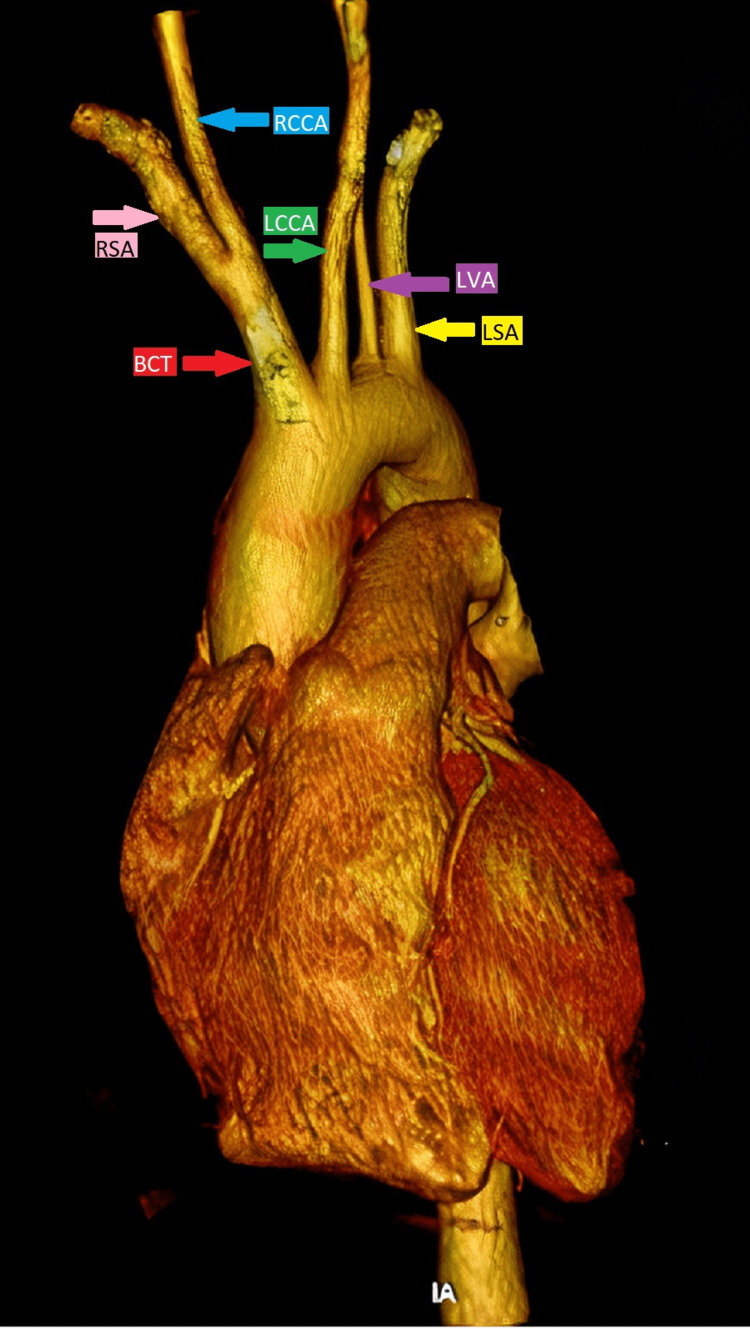
CTA image of arcus aorta and supra-aortic branches belonging to 4b1 subgroup CTA: computed tomography angiography; RSA: right subclavian artery; RCCA: right common carotid artery; LCCA: left common carotid artery; LSA: left subclavian artery; BCT: brachiocephalic trunk; LVA: left vertebral artery

Four branches, BCT, LCCA, LVA, and LSA, emerge from the AA. This type of variation was identified in a total of five (0.7%) patients (four males, one female) (Figure 12).

4b5

**Figure 13:**
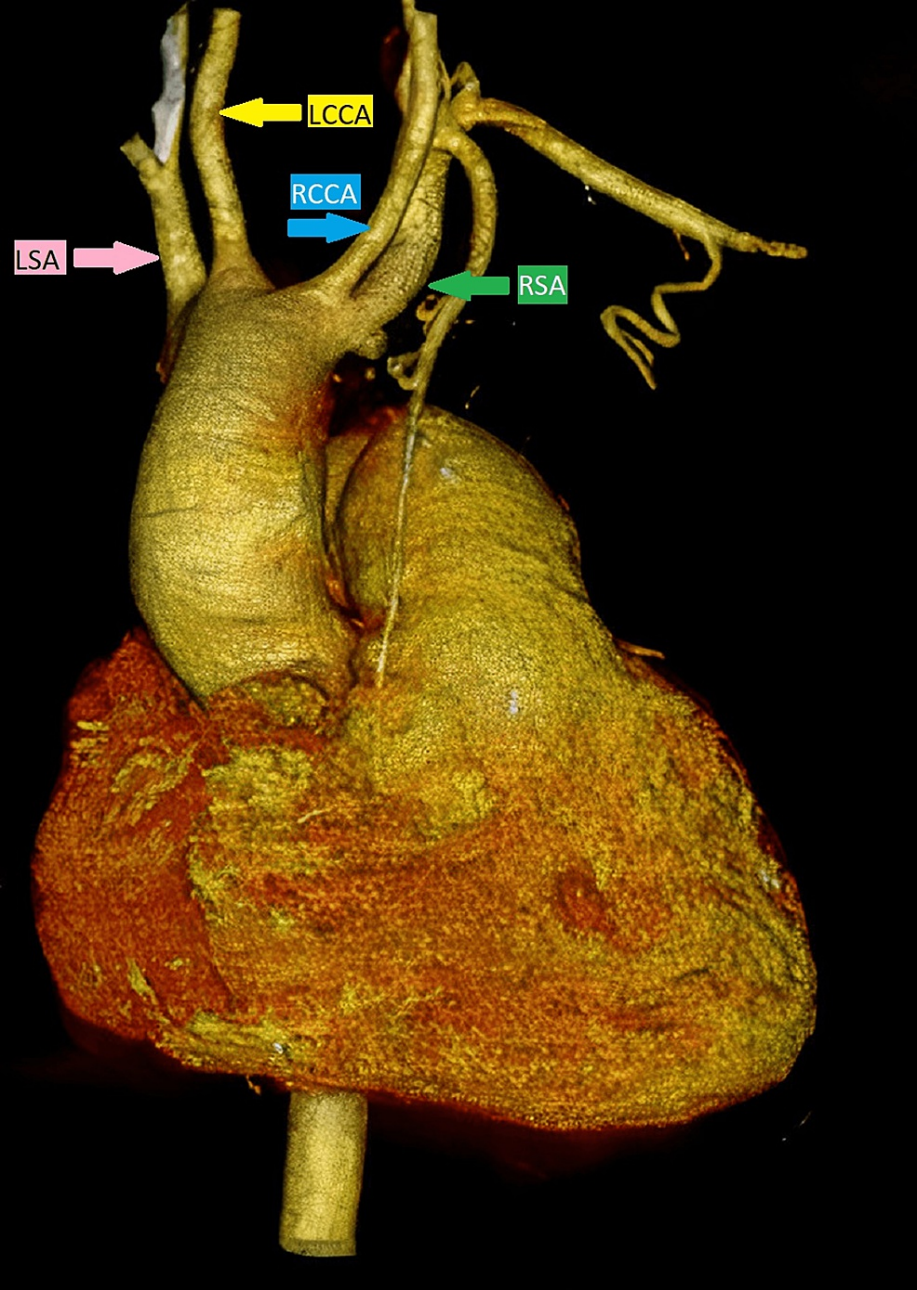
CTA image of arcus aorta and supra-aortic branches belonging to 4b5 subgroup CTA: computed tomography angiography; RSA: right subclavian artery; RCCA: right common carotid artery; LCCA: left common carotid artery; LSA: left subclavian artery

RSA, RCCA, LCCA, and LSA arise from the AA as separate branches. This branching pattern was observed in only one (0.1%) patient (female) (Figure 13). The AA branching variations identified in this study and their descriptions are shown in Table 1.

**Table 1:** Types and frequencies of the branches arising from the aortic arch CT: common trunk; RSA: right subclavian artery; RCCA: right common carotid artery; LCCA: left common carotid artery; LSA: left subclavian artery; BCT: brachiocephalic trunk; LVA: left vertebral artery

Number of branches	Branching subtype	Description	Number	Percentage
1	1b1	CT (RSA- RCCA- LCCA- LSA) branching off from AA	1	0.1%
2	2b1	CT (RSA- RCCA- LCCA) from the right side, LSA from the left side	168	24%
	2b2	BCT and LCCA emerging from a common ostium on the right and LSA on the left	30	4.3%
	2b3	BCT and CT (LCCA-LSA)	11	1.6%
3	3b1	BCT-LCCA-LSA from the right side	479	68.5%
	3b2	BCT-LVA-LSA from the right side	1	0.1%
	3b7	BCT-LCCA-CT (LVA-LSA)	3	0.4%
4	4b1	BCT- LCCA- LVA- LSA from the right side	5	0.7%
	4b5	RSA, RCCA, LCCA, LSA	1	0.1%

When we analyzed the relationship of these variations with sex, a statistically significant difference was observed (p<0.05). Type 3b1 (the normal branching pattern) was more common in males, whereas other branching patterns, designated as variations, were predominantly observed in females. The sex distribution of the patients with anatomical branching variations is presented in Table 2.

**Table 2:** Distribution of anatomical variations in the aortic arch branching pattern by sex

Branching type	Male, N (%)	Female, N (%)	Total, N (%)
1b1	0	1 (0.1%)	1 (0.1%)
2b1	60 (8.6%)	108 (15.5%)	168 (24%)
2b2	9 (1.3%)	21 (3.0%)	30 (4.3%)
2b3	2 (0.3%)	9 (1.3%)	11 (1.6%)
3b1	244 (34,9%)	235 (33.6%)	479 (68.5%)
3b2	1 (0.1%)	0 (0.0%)	1 (0.1%)
3b7	0 (0.0%)	3 (0.4%)	3 (0.4%)
4b1	4 (0.6%)	1 (0.1%)	5 (0.7%)
4b5	0 (0.0%)	1 (0.1%)	1 (0.1%)
Total	320 (45.8%)	379 (54.1%)	699

## Discussion

The normal AA branching pattern has been reported at prevalence rates ranging from 60% to 99% in studies on different populations [1-4,7,14-17]. In this study, the usual AA branching pattern (type 3b1) was found in 68.5% of the patients. The prevalence rate of 68.5% as demonstrated in this study is consistent with previous reports.

In this study, the other eight branching patterns, collectively designated as the aortic arch variations, were detected in 31.5% of the patients. While the reported prevalence of the normal AA branching pattern is above 80% in Japan, China, Australia, Poland, and India, it is less than 70% in the United States, Kenya, and Jordan [1,4,13]. In a study examining 4000 images from patients from Southern India, AA variations were reported at a rate of 0.675%, whereas a study from Jordan involving 500 patient images reported a prevalence of 38.8% [1,14]. We think that this wide range of reported frequencies can be related to genetic and racial differences among the populations studied [5,15]. However, although the prevalence rate that we found in this study is in line with the literature data [1,4,13,14,18], it is higher than the variation rates reported by studies in the Turkish population [2,7,10,15,19]. In order to better understand the cause of this discrepancy, a large-scale, multicenter study across different regions of Turkey is warranted. The frequency distribution of the variations in aortic arch branching pattern as found in our study is presented in Table 3 along with those reported by published studies.

**Table 3:** Prevalence rates of aortic arch branching patterns from previous studies and the current study CTA: computed tomography angiography; CT: computed tomography; DSA: digital subtraction angiography

Study	Country	1b1	2b1	2b2	2b3	3b1	3b2	3b7	4b1	4b5
Mustafa et al., 2017 [1] (500 CTA)	Jordan	-	-	156 (31.2%)	1 (0.2%)	306 (61.2%)	6 (1.2%)	1 (0.2%)	22 (4.4%)	-
Açar et al., 2022 [2] (1026 CT)	Turkey	-	100 (9.7%)	81 (7.9%)	1 (0.097%)	781 (76.12%)	9 (0.88%)	1 (0.097%)	37 (3.6%)	1 (0.097%)
Kumar and Mishra, 2015 [3] (42 cadavers)	Nepal	-	1 (2.38%)	-	-	35 (83.3%)	-	-	5 (11.9%)	1 (2.38%)
Budhiraja et al., 2013 [6] (52 cadavers)	India	-	10 (19.2%)	-	-	33 (63.5%)	1 (1.9%)	-	8 (15.3%)	-
Ergun et al., 2015 [7] (270 DSA)	Turkey	-	58 (21.5%)	-	-	198 (73.3%)	3 (1.1%)	-	7 (2.6%)	-
Celikyay et al. 2013 [10] (1361 CTA)	Turkey	-	240 (21.1%)	-	-	845 (74.4%)	8	-	33	1
Natsis et al., 2021 [13]	review	1	626 (9%)	159 (2%)	24	3515 (78%)	27 (1%)	1	161 (%4)	2
Karacan et al., 2014 [15] (1000 CTA)	Turkey	-	141 (14.1%)	-	-	792 (79.2%)	12 (1.2%)	-	41 (4.1%)	-
Aboulhoda et al., 2019 [16] (100 CT)	Egypt	-	24 (24%)	6 (6%)	-	65 (65%)	-	-	5 (%5)	-
Lale et al., 2014 [19] (881 CTA)	Turkey	-	64 (7.2%)	-	-	770 (87.4%)	-	-	25 (28%)	-
Tapia et al., 2015 [20] (1050 CT)	China	-	-	139 (13.23%)	-	853 (81.23%)	1 (0.09%)	-	-	-
Wang et al., 2016 [21] (2370 CT)	China	-	-	229 (9.6%)	-	1985 (83.8%)	14 (0.6%)	-	112 (%4.7)	-
Tapia-Nañez et al., 2021 [22] (220 CTA)	Mexico	-	30 (13.6%)	-	-	171 (77.7%)	1 (0.5%)	-	16 (7.3%)	-
O'Malley et al., 2018 [23] (24 cadavers)	Ireland	-	2 (12.5%)	-	-	19 (79.2%)	-	-	2 (8.3%)	-
Vučurević et al., 2013 [24] (1266 CT, CTA)	Serbia	-	84 (6.64%)	86 (6.79%)	36 (2.84%)	946 (74.72%)	-	-	46 (%3.63)	3 (0.24%)
Current study (699 CTA)	Turkey	1 (0.1%)	168 (24%)	30 (4.3%)	11 (1.6%)	479 (68.5%)	1 (0.1%)	3 (%0.4)	5 (0.7%)	1 (0.1%)

While the type 1b1 variation (branching of RSA, RCCA, LCCA and LSA from a common trunk) was observed in only one (0.1%) patient in this study, Natsis et al. reported this variation at a frequency of 1% [13].

The variation types 2b1 and 2b2 have been designated as bovine arch clinically and in the literature. Although there are studies arguing that the term “bovine arch” is a misnomer [7], we used this generally accepted term to identify these two variations [2,8]. Bovine arch is the second most common branching variation after the usual branching pattern [1,2,8,13,15,20,21]. In a study reviewing 4000 CT images, Pandalai et al. observed bovine arch configuration (i.e., types 2b1 and 2b2) in only one patient [14]. However, Dumfarth et al. reported that the bovine arch was the most common variation, with a prevalence of 24.6% [8]. In a study investigating aortic arch branching patterns in Chinese patients with or without aortic dissection, the bovine arch was the most prevalent type (13.23%) in both groups, after the normal branching pattern [20]. In our study, the combined frequency of the variation types 2b1 and 2b2 representing the bovine arch variation was 28.3%, which is consistent with the literature. There are studies reporting that individuals with the bovine arch branching pattern have a 50% chance of developing infarction and that this abnormal AA configuration presents technical difficulties in endovascular procedures in this region [9]. It has been reported that in patients with bovine arch variation, carotid artery stenting can be performed more easily via the brachial or radial artery rather than the femoral artery due to angulation of the vessels [25]. Moreover, this branching pattern has been reported to be associated with the occurrence of aneurysms or type B aortic dissection [20,26].

In a comprehensive review, Natsis et al. reported that the AA variation classified in this study as type 2b3 (two branches arising from the aortic arch) at an incidence of 2% [13]. This variation was reported at a rate of 0.2% in the Jordanian population [1] and 0.097% in the Turkish population [2]. In our study, 1.6% of the patients was found to have type 2b3, which is in line with Natsis et al.’s findings.

The prevalence rates of the type 3b2 variation were 0.88% as reported by Açar et al., 1.2% by Karacan et al., 1.1% by Ergun et al. 2% by Natsis, 1.2% by Berko et al., 1.9% by Budhiraja et al. and 0.6% by Wang et al. [2,6,7,11,13,15,17,21]. Trubert et al. observed this variation in 1 out of 33 patients undergoing total thoracic aorta endovascular repair [2,6,7,11,13,15,17,21]. The type 3b2 variation was reported in 1 of 4000 patients in the Indian population and 1.2% of the Jordanian population versus 0.1% in this study [1,14]. Origination of the vertebral artery directly from the aortic arch is important in endovascular interventions for the diagnosis and treatment of posterior cerebral events [16,27]. The type 3b7 variation was reported by Natsis et al. in only one patient [13], and Mustafa et al. found this aberrant configuration in two (0.4%) individuals [1].

The presence of vertebral artery directly originating from the aortic arch was observed in 4.85% of a sample of 1050 patients, including 525 patients diagnosed with aortic dissection and 525 control subjects [20]. This AA variation (type 4b1) was reported at a prevalence of 23% by Natsis et al. [13]. The type 4b1 branching pattern was reported at rates of 6% (Jordanian population), 11.9% (Nepal population), 4.7% (Chinese population), 3.697% by Açar et al., 4.1% by Karacan et al., and 2.6% by Ergun et al. in the Turkish population, and 4.2% in patients with congenital heart disease versus 2.7% in controls by Tawfik et al., 7.3% by Tapia-Nanez et al., 2.8% by Popieluszko et al., 8.3% by O’Malley et al., and 15.3% by Budhiraja et al. [1-4,6,7,15,21-23,28]. While this type was the second or third most common AA variation in other studies, it ranked fourth among all types of variations in our study, although its prevalence was lower. Gluncic et al. reported that anomalous origin of the vertebral artery may be associated with hemodynamic changes, which could lead to atherosclerosis and other cerebral disorders [29].

Another type of variation was the type 4b5 branching pattern where the right and left subclavian arteries and right and left common carotid arteries originate separately from the aortic arch. This atypical branching pattern was identified in only one patient (0.1%) in our study versus 0.097% (n=1) as reported by Açar et al. and 1% (n=2) by Natsis et al. [2,13].

In a study, Dumfarth et al. suggested that all aortic arch variations should be regarded as risk factors for aortic disease [8]. It has been reported that both common carotid arteries originating together as a single trunk or the vertebral artery emerging from a different origin are associated with an increased incidence of cerebrovascular diseases [24].

Limitations

The limitations of this study included it is carried out in a single center, CTAs with good image quality are selected, and other imaging methods are not being used.

## Conclusions

Aortic arch branching patterns can cause hemodynamic changes, leading to atherosclerosis and other cerebrovascular pathologies. Detection of variations in aortic arch branching patterns with CTA before interventions to the aortic arch will allow shortening the procedure time, reducing the amount of contrast material used, and choosing the appropriate stent. Therefore, specialists planning surgical or endovascular interventions in this region should know and have knowledge of atypical aortic branching patterns.

Higher prevalence rates of AA branching patterns compared to previous studies were identified in the Turkish population in this study and therefore, a comprehensive, multicenter study built on our data would be needed. We think that the Horos software (an open-source image viewer) can be used reliably in diagnostic procedures, as it enables 3D assessment of the vessels on CTA images.
